# The value of lymphocyte-to-monocyte ratio and neutrophil-to-lymphocyte ratio in differentiating pneumonia from upper respiratory tract infection (URTI) in children: a cross-sectional study

**DOI:** 10.1186/s12887-021-03018-y

**Published:** 2021-12-03

**Authors:** Jinghua Wu, Xu Wang, Mingqi Zhou, Guo-Bo Chen, Jing Du, Ying Wang, Chengyin Ye

**Affiliations:** 1 Department of health management, Hangzhou Normal University, Zhejiang, 310000 Hangzhou China; 2 Engineering Research Center of Mobile Health Management System, Ministry of Education, Hangzhou Normal University, Zhejiang, 310000 Hangzhou China; 3 Department of Laboratory Medicine, Zhejiang Provincial People’s Hospital, People’s Hospital of Hangzhou Medical College, Zhejiang, 310014 Hangzhou China; 4 School of Laboratory Medicine and Life Science, Wenzhou Medical University, Wenzhou, Zhejiang, 325035 China; 5 Key Laboratory of Endocrine Gland Diseases of Zhejiang Province, Zhejiang, 310000 Hangzhou China; 6 Phase I Clinical Research Center, Zhejiang Provincial People’s Hospital, Affiliated People’s Hospital, Hangzhou Medical College, Zhejiang, 310000 Hangzhou China

**Keywords:** Pneumonia, Upper respiratory tract infection, Neutrophil-to-lymphocyte ratio, Lymphocyte-to-monocyte ratio

## Abstract

**Backgrounds:**

Early and accurate diagnosis of pediatric pneumonia in primary health care can reduce the chance of long-term respiratory diseases, related hospitalizations and mortality while lowering medical costs. The aim of this study was to assess the value of blood biomarkers, clinical symptoms and their combination in assisting discrimination of pneumonia from upper respiratory tract infection (URTI) in children.

**Methods:**

Both univariate and multivariate logistic regressions were used to build the pneumonia screening model based on a retrospective cohort, comprised of 5211 children (age ≤ 18 years). The electronic health records of the patients, who had inpatient admission or outpatient visits between February 15, 2012 to September 30, 2018, were extracted from the hospital information system of Zhejiang Provincial People’s Hospital, Hangzhou, Zhejiang Province, China. The children who were diagnosed with pneumonia and URTI were enrolled and their clinical features and levels of blood biomarkers were compared. Using the area under the ROC curve, both two screening models were evaluated under 80% (training) versus 20% (test) cross-validation data split for their accuracy.

**Results:**

In the retrospective cohort, 2548 of 5211 children were diagnosed with the defined pneumonia. The univariate screening model reached predicted AUCs of 0.76 for lymphocyte/monocyte ratio (LMR) and 0.71 for neutrophil/lymphocyte ratio (NLR) when identified overall pneumonia from URTI, attaining the best performance among the biomarker candidates. In subgroup analysis, LMR and NLR attained AUCs of 0.80 and 0.86 to differentiate viral pneumonia from URTI, and AUCs of 0.77 and 0.71 to discriminate bacterial pneumonia from URTI respectively. After integrating LMR and NLR with three clinical symptoms of fever, cough and rhinorrhea, the multivariate screening model obtained increased predictive values, reaching validated AUCs of 0.84, 0.95 and 0.86 for distinguishing pneumonia, viral pneumonia and bacterial pneumonia from URTI respectively.

**Conclusions:**

Our study demonstrated that combining LMR and NLR with critical clinical characteristics reached promising accuracy in differentiating pneumonia from URTI, thus could be considered as a useful screening tool to assist the diagnosis of pneumonia, in particular, in community healthcare centers. Further researches could be conducted to evaluate the model’s clinical utility and cost-effectiveness in primary care scenarios to facilitate pneumonia diagnosis, especially in rural settings.

**Supplementary Information:**

The online version contains supplementary material available at 10.1186/s12887-021-03018-y.

## Introduction

Pneumonia is the leading cause of hospitalizations and death among children globally. In 2015, the estimated cause-specific mortality rate of pneumonia was 5.455 cases per 1000 live births [1]. Therefore, childhood pneumonia causes a significant burden on both patients and their families, including substantial expenses, loss of routine, and decrease in quality of life [2]. Furthermore, pneumonia in early childhood has increasingly been associated with reduced lung function and the development of chronic non-communicable respiratory diseases, such as asthma or chronic obstructive pulmonary disease, both in children and adults [3–6].

Although chest X-ray is considered as a critical step for pneumonia diagnosis with high accuracy, it still has some shortcomings, including high expense, unnecessary check and inconsistency in radiographies by physicians [7]. Accurate diagnosis of pneumonia in primary care remains difficult as it is impractical to send all children to chest X-rays examinations, most primary physicians therefore initially rely on clinical signs and blood routine test [8]. However, due to some overlaps of symptoms between pneumonia and upper respiratory tract infection (URTI), such as fever and cough, it is still a challenge to identify children infected by pneumonia from the patients with URTI, especially for those primary healthcare workers who lack of expertise in diagnosing suspected pneumonia cases or in some resource-poor primary care settings where chest X-ray is unavailable [9, 10].

As one of the most practical ways to track patients’ physical condition, blood routine test usually measures the levels of neutrophils cell (NC), monocytes cell (MC), lymphocyte cell (LC), white blood cell count (WBC) and C-reactive protein (CRP) in blood. Among them, neutrophils, lymphocytes and monocytes are common indicators of human body’s inflammation and immune status. Microbes, such as respiratory syncytial virus, influenza virus, pneumococcus, or *staphylococcus aureus*, were generally detected in patients with pneumonia [11–13], and the rapid accumulation of neutrophils is recognized as the key to effectively clean up microbe threats [14]. Other leukocytes, including monocytes and lymphoid cells, could also be recruited in such antimicrobial immune process [15]. However, in addition to pneumonia, the levels of these biomarkers may also be affected by other factors, such as leukemia, acute infection and tumor, showing great variations among individuals [16–18]. Nevertheless, the calculated neutrophil/lymphocyte ratio (NLR) and lymphocyte/monocyte ratio (LMR) can eliminate such variation and be sensitive to reflect the balance between inflammatory response and immune status in patients [19]. Studies have shown that NLR and LMR are good indicators in evaluating prognosis of various diseases, such as malignant tumors, etc. [20]. With the capacity of notifying inflammatory response and immune status in patients, we wonder whether NLR or LMR could be used as preliminary indicators to screen children at high risk of pneumonia and to help determine the needs for further chest X-ray examinations, as well as to identify children at low risk of pneumonia and avoid unnecessary chest X-ray checks for these low-risk children.

In this study, we aimed to develop a clinical primary screening tool to differentiate pneumonia from URTI, by using children’s EHR data in Zhejiang Provincial People’s Hospital, Hangzhou, Zhejiang Province, China. We anticipate the tool can help clinicians make clinical decisions about who should be sent for chest radiography examination to identify possible pneumonia, and facilitate precise diagnosis by reducing unnecessary medical expenses.

## Methods

### Cohort

According to the corresponding inclusion and exclusion criteria described below, a total of 5211 eligible patients that have inpatient admission or outpatient visits to Zhejiang Provincial People’s Hospital from February 15, 2012 to September 30, 2018, were enrolled in this retrospective study cohort. In this retrospective observational study, the use of the de-identified data was authorized, patients having diagnostic records of URTI, viral or bacterial pneumonia, were extracted from the EMR big data intelligent platform of Zhejiang Provincial People’s Hospital information system, along with their demographics, laboratory test results, chest x-ray records, and clinical symptoms information at the time of their initial inpatient admission or outpatient visits. All personal privacy information was well protected and removed during the analysis and publication process. This study was approved by ethics committee of Zhejiang Provincial People’s Hospital (No. 2021QT222), and was exempt from informed consent as shown by the IRB approval letter. Since this was a diagnostic accuracy study, we followed the Standards for Reporting of Diagnostic Accuracy Studies (STARD) and completed the STARD checklist (see Supplementary Table 1) [21].

**Fig. 1:**
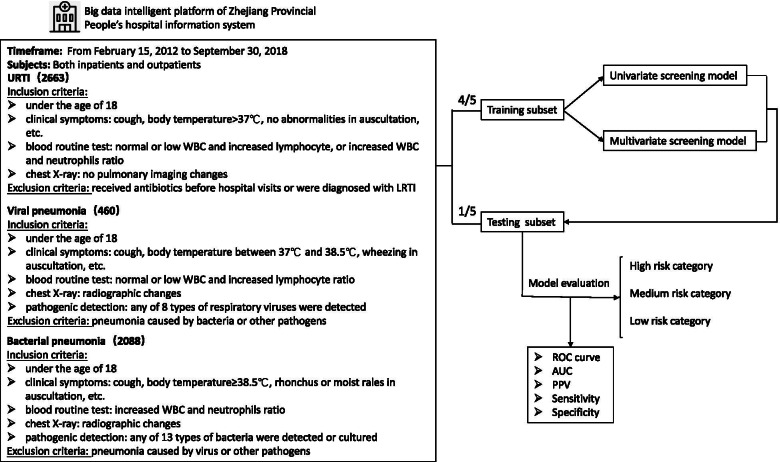
Study design

The inclusion and exclusion criteria were demonstrated carefully in the study design workflow (Fig. 1), and the detailed diagnostic criteria of URTI and pneumonia were summarized in Supplementary Table 2. Specifically, patients with URTI were defined as those who meet all of the following criteria: 1) under the age of 18 years, 2) having clinical signs such as cough, swollen and congested tonsils, hyperemia, edema, and secretions in nasal mucosa or pharynx, or runny nose, body temperature > 37 °C, and no abnormalities in lung auscultation, 3) having normal or low WBC and increased lymphocyte ratio that indicates viral infections, or increased WBC and neutrophils ratio that implies bacterial infections, 4) showing no pulmonary imaging changes in chest X-ray if any. Those who had received antibiotics before hospital visits or were diagnosed with pneumonia, bronchitis, and other lower respiratory tract infections at hospital were excluded from the URTI cohort. Patients with viral pneumonia were defined as children who meet all of the following criteria during their hospital visits: 1) under the age of 18 years, 2) having clinical symptoms of cough, body temperature between 37 °C and 38.5 °C generally, wheezing in auscultation, tachypnoea, breathlessness, or chest pain, etc., 3) having normal or low WBC and increased lymphocyte ratio, 4) showing multifocal 1–10-mm well-defined or ill-defined nodular opacity with a surrounding halo or patchy ground-glass opacity (GGO), or other clues of viral pneumonia in the chest radiograph, as summarized in Supplementary Table 2, 5) having any of 8 types of respiratory viruses detected from nasopharyngeal or throat swabs, including respiratory syncytial virus (RSV), adenovirus nucleic acid (ADVDNA), influenza A virus antigen (FluA-Ag), and so on. Patients diagnosed with pneumonia caused by bacteria or other pathogens were excluded from the viral-pneumonia cases. Patients with bacterial pneumonia were defined as those meet all of the following criteria: 1) under the age of 18 years, 2) having clinical symptoms of cough, body temperature ≥ 38.5 °C generally, rhonchus or moist rales in lung auscultation, tachypnoea, breathlessness, or chest pain, etc., 3) having increased WBC and neutrophils ratio, 4) showing patchy shadow, lung consolidation (usually lobed or segmentary with bronchial inflation), centrilobular lung nodules (Solid or mixed density nodules along the bronchovascular bundle), or other signs of bacterial pneumonia in the chest radiograph, as summarized in Supplementary Table 2, 5) having any of 13 types of bacteria detected or cultured from nasopharyngeal swabs, sputum, alveolar lavage fluid, pleural effusion or fiberoptic bronchoscopy smear, such as *pseudomonas aeruginosa*, candida albicans, mixed flora, klebsiella pneumoniae, and so on. Those who diagnosed with pneumonia caused by virus or other pathogens were excluded from the bacterial-pneumonia cases. Since the data of this retrospective cohort study was extracted from the big data platform, only those having complete diagnostic information, clinical symptoms or signs, blood routine examination and having chest X-ray results when diagnosed with pneumonia were included for analysis.

### Specimen collection and laboratory procedures

As a routine blood test for children presenting to the facility, the complete blood count was conducted on each patient to measure the concentration of neutrophils cell (NC), monocytes cell (MC), lymphocyte cell (LC), white blood cell count (WBC), C-reactive protein (CRP). Pleural effusion puncture was performed under ultrasound guidance so as to extract pleural effusion from chest cavity. Microscopes, culture medium or polymerase chain reaction (PCR) were used to detect pathogenic bacteria. In accordance to the standard sample collection procedures, nasopharyngeal or throat swabs were collected to detect respiratory viruses or bacteria. Sputum was cultured for anaerobes, aerobes, fungus, as well as mycobacterium tuberculosis, in order to diagnose the causes of respiratory infection. To obtain fiberoptic bronchoscopy smear, the fiberoptic bronchoscopy was brushed twice at the suspicious site under tracheal mirror positioning and the entrapped cells were smeared and sent for examination. The alveolar bronchus was repeatedly rinsed with normal saline to obtain alveolar lavage fluid. The fluid was further centrifuged at 2000r / min for 3 min, where the supernatant was discarded and the sediment was smeared.

### Statistical analysis

Categorical variables were compared using chis-square and continuous variables were assessed using Mann–Whitney U test. The sample.split function from package caTools of R libraries was applied to split the study cohort randomly into two subsets at the predefined ratio. In our study, the retrospective cohort (5211 patients) was split into the training and testing subsets at a ratio of 4:1, where the number of pneumonia and URTI cases was uniformly distributed across the training and testing subsets. The modeling process was implemented in two phases: (1) the training subset was used to develop the model and generate predictive estimates and (2) then was validated in the testing subset. First, we built the univariate screening model by treating blood biomarkers (WBC, NC, MC, LC, CRP, LMR, NLR) independently as discriminators. Among them, NLR was calculated as the ratio of neutrophils to lymphocytes while LMR was calculated as the ratio of lymphocytes to monocytes. Secondly, we built the multivariate screening model by combining symptoms of age, fever, cough and rhinorrhea (RHI), and the most significant biomarkers (NLR and LMR) all together as pneumonia indicators. For both univariate and multivariate model, each individual’s risk score in either training or validation cohort was calculated firstly. Then, individuals in training set were sorted by their risk scores from low to high, and the 25 and 75% quantiles were obtained as cut-offs to divide individuals into three risk categories. After that, these determined cut-offs were applied to the validation cohort, thereafter, three risk categories of the validation set were captured, indicating the high, medium, and low risks of pneumonia. Following that, positive predictive values (PPVs), sensitivity and specificity were carefully calculated. A subgroup analysis was also performed to evaluate the above built models for distinguishing two different types (bacterial and viral) of pneumonia from URTI. The area under the ROC curve was used to evaluate the accuracy of the screening model. All statistical analyses were conducted in R software version 3.6.1. R libraries, including glm, ROCR, AUC, ggplot and pROC, were applied respectively.

## Results

### Baseline characteristics

A total of 5211 children were included in our study. Among them, 2548 (48.9%) were diagnosed with pneumonia and 2663 (51.1%) were with URTI. Baseline demographic features, clinical features, as well as the blood inflammatory markers, were summarized in Table 1. Age showed statistical difference between the two sub-cohorts (*p* < 0.05), the overall distribution of children with pneumonia was balanced in all age groups, while the number of children with URTI generally increases with age, with only 9.76% of URTI population younger than 1-year-old and 43.94% older than 7 years of age. Gender showed no difference between the two sub-cohorts (*p* > 0.05), with the male patients occupying 54.3% of pneumonia and 53.44% of URTI patients respectively. Three major clinical symptoms showed statistical difference between the two sub-cohorts, where the frequency of fever (defined as temperature ≥ 37.0 °C) and coughing was higher in patients with pneumonia, while more rhinorrhea individuals were observed in URTI sub-cohort. Most blood biomarkers, except WBC, all showed statistical difference between the two sub-cohorts (*p* < 0.05).

**Table 1:** Distribution of baseline features in children diagnosed with pneumonia and URTI

Parameter	Pneumonia	URTI
	(*N* = 2548)	(*N* = 2663)
Demographic, n (%)		
Age (years)*		
< 1	741 (29.08)	260 (9.76)
1–2	528 (20.72)	434 (16.3)
3–4	484 (19)	443 (16.64)
5–6	310 (12.17)	356 (13.37)
≥ 7	485 (19.03)	1170 (43.94)
Male sex	1384 (54.32)	1423 (53.44)
Clinical features, n (%)		
Fever (BT ≥ 37 °C) *	1967 (77.2)	1705 (64.03)
Cough *	2365 (92.82)	1594 (59.86)
Rhinorrhea *	561 (22.02)	859 (32.26)
Complete blood count, median (IQR)		
WBC (×10^9/L)	7.66 (5.91, 10.06)	7.78 (5.87,10.22)
NC (×10^9/L) *	3.03 (1.92,4.63)	4.44 (2.83,6.7)
MC (×10^9/L) *	0.49 (0.34,0.71)	0.6 (0.45,0.78)
EC (× 10^9/L) *	0.14 (0.05,0.27)	0.07 (0.02,0.18)
LC (×10^9/L) *	3.4 (2.22,5.11)	2.07 (1.34,3.19)
CRP (mg/L) *	3.8 (1.2,9.6)	4 (1.5,9.8)
LMR*	7.09 (4.57,10.61)	3.45 (2.14,5.61)
NLR*	0.87 (0.43,1.76)	2.18 (1.09,4.27)

For the comparison between pneumonia and UTRI, Chi-square test for categorical variables and Wilcoxon-rank sum test for continuous variable was employed; *p < 0.05

### Model performance

**Fig. 2:**
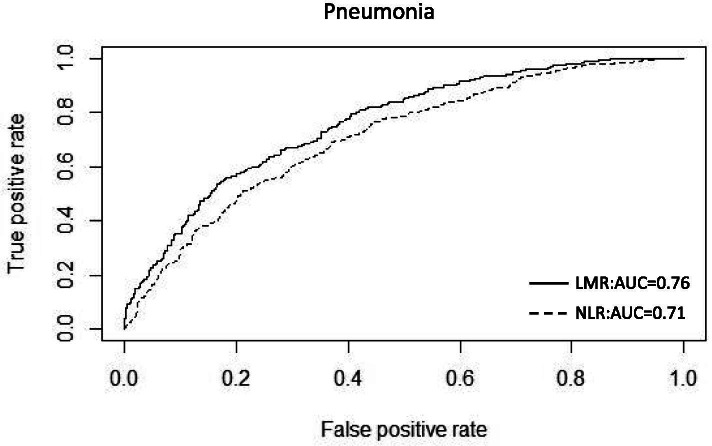
The ROC curves for LMR and NLR to differentiate pneumonia from URTI on the validation set

**Fig. 3:**
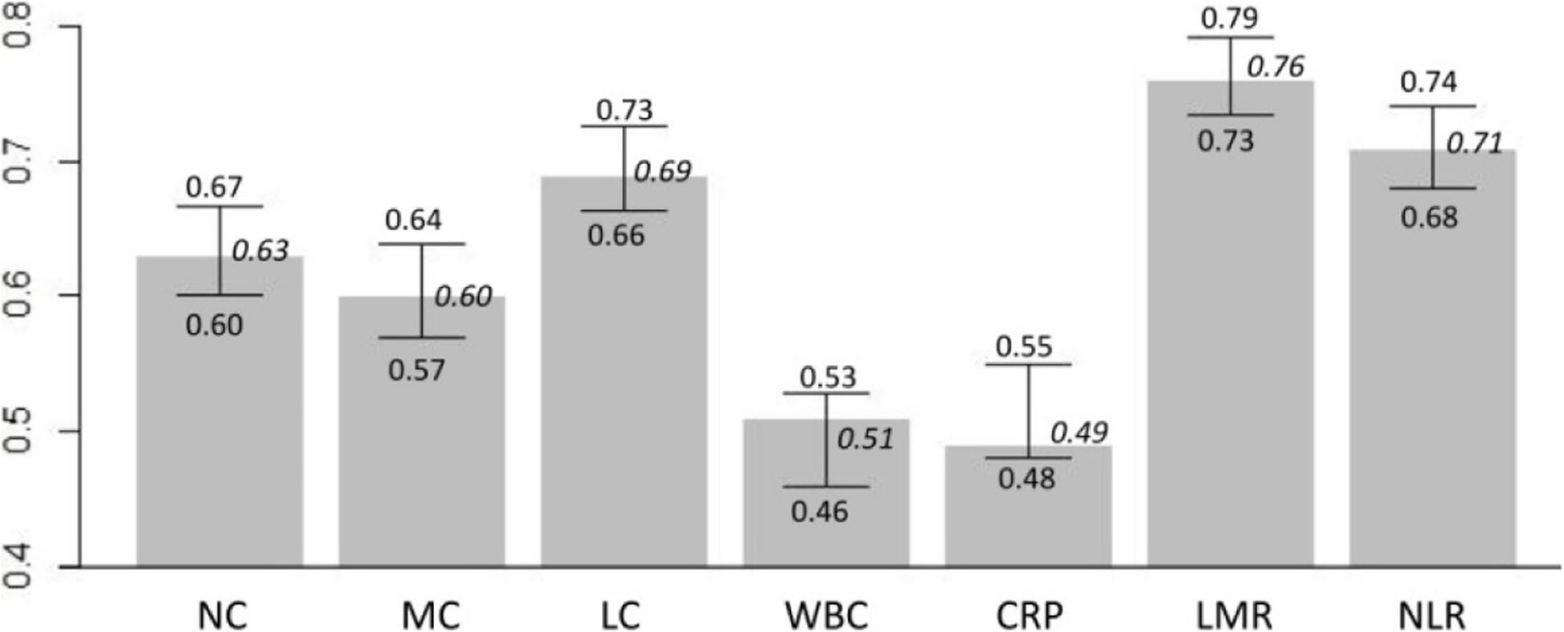
The validated AUC values of each blood biomarkers and 95% CI to identify pneumonia against URTI

Initially, we divided the data into training and testing sets at a ratio of 4:1 to build the univariate screening model, and evaluated the model’s performance on an independent testing data. In summary, LMR and NLR showed better validated discriminative accuracy than other biomarkers (LC, NC, MC, WBC and CRP), either in distinguishing the overall pneumonia from URTI or in discriminating subgroups of viral or bacterial pneumonia from URTI. LMR attained the highest accuracy among all biomarkers mentioned above, reaching a predicted AUC of 0.76 (95% CI: 0.73–0.79) on the validation set to distinguish the overall pneumonia from URTI (Fig. 2). NLR also achieved a relatively high validated AUC of 0.71 (95% CI: 0.68–0.74). On the contrary, while introducing other blood biomarkers into the validation procedure, they all achieved relatively low AUC values (range 0.49–0.69), showing little discriminative values (see Supplementary Table 3). More details about the validated AUCs of each blood biomarkers and their 95% confidence intervals were shown below (Fig. 3). In the subgroup analysis to distinguish two different types (viral or bacterial) of pneumonia and URTI, the two indicators, LMR and NLR, also demonstrated their promising performance better than all other biomarkers, as they attained predicted AUCs of 0.86 (95% CI: 0.82–0.90) and 0.80 (95% CI: 0.76–0.84) in distinguishing viral pneumonia from URTI respectively, and achieved the AUCs of 0.77 (95% CI: 0.74–0.80) and 0.71 (95% CI: 0.67–0.74) in differentiating bacterial pneumonia against URTI (see Supplementary Fig. 1).

Recognized as the two strongest predictors of pneumonia in children, LMR was found to increase the risk of viral, bacterial and combined pneumonia when considering URTI as reference, and it attained ORs of 1.24, 1.26 and 1.27 respectively in our study. In contrast, NLR decreased the risk of three different types of pneumonia, with ORs of 0.23, 0.73 and 0.67 respectively (see Supplementary Table 4).

**Fig. 4:**
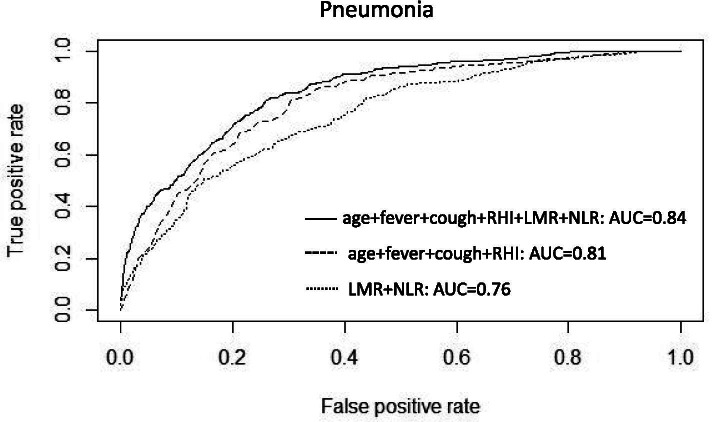
The ROC curve of three different models to discriminate pneumonia against URTI

Since LMR and NLR were validated as valuable predictors of pneumonia, reaching relatively high classification accuracy individually, we also investigated whether the integration of the two indicators with other important clinical symptoms could further construct a multivariate screening model with improved accuracy. The results showed that, when combining age, clinical symptoms (i.e., fever, cough and rhinorrhea) together with LMR and NLR, the screening model reached an AUC of 0.84 (95% CI: 0.82–0.87) for discriminating the overall pneumonia from URTI, better than the model considering LMR and NLR (AUC = 0.76[0.73–0.78]) only or the model using clinical symptoms and age (AUC = 0.81[0.78–0.83]) alone (see Fig. 4). It is worth noting that in the subgroup analysis for identifying viral pneumonia from URTI, the integrated model reached an AUC value as high as 0.95 (95% CI: 0.93–0.97), outperformed than the other two compared models. As for identifying bacterial pneumonia from URTI, the integrated model reached an AUC of 0.86 (0.83–0.88). We further investigated whether the integration of LMR and NLR with other important clinical symptoms could construct a multivariate screening model to differentiate bacterial pneumonia from viral pneumonia. The results showed that, when combining age, clinical symptoms (i.e., fever, cough and rhinorrhea) together with LMR and NLR, the integrated screening model reached an AUC of 0.83 (0.80–0.87), still slightly better than the model considering LMR and NLR (AUC = 0.75) only and the model using clinical symptoms and age (AUC = 0.82) alone. The details were all carefully introduced in Supplementary Fig. 2.

Besides, several studies have illustrated that CRP could be used to assist pneumonia diagnosis [22], thus we consequently combined CRP, age and clinical signs to build a new multivariate screening model and compared its discriminating ability with our integrated model using LMR and NLR. The results showed that, although the CRP-involved multivariate model attained a relatively high validated AUC value of 0.81 (95% CI: 0.79–0.84) in distinguishing the overall pneumonia from URTI, it was still lower than our multivariate screening model recruiting LMR and NLR. The relatively high performance of the CRP-involved model was indeed attributed to the good screening ability of included clinical signs. Subgroup (viral and bacterial) analysis also indicated similar result that our integrated model achieved better discriminating ability than the CRP-involved model, which was carefully demonstrated in Supplementary Fig. 3.

**Fig. 5:**
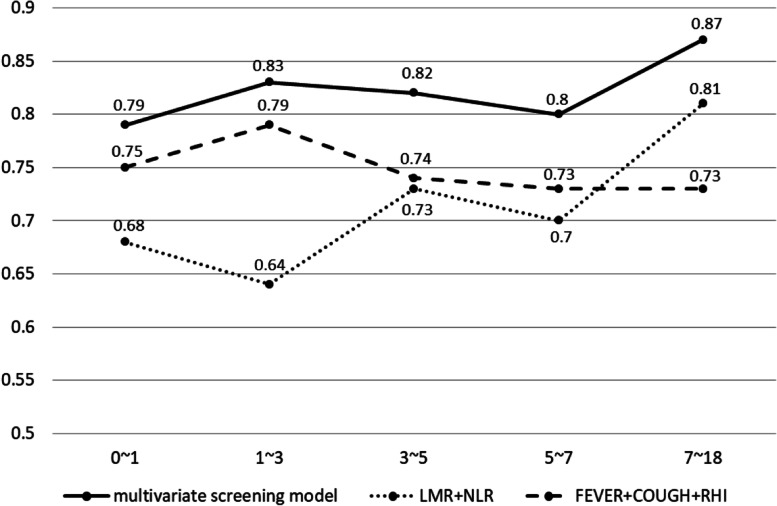
The performance of three models at different age stratifications

Considering that age profoundly affects incidence and development of most pediatric diseases, we also explored the influence of age on the model’s performance and revealed the model’s capability across various age stratifications. As the results showed, the multivariate screening model attained better performance in all age groups, compared to the other two models, with its best performance at 1–3 (AUC:0.83) and over 7 (AUC:0.87) age groups. After calculating the PPVs, sensitivity and specificity for the identified high-risk category within various age stratifications, it was found that the built multivariate screening model reached steady and relatively high PPVs across all age groups, with all values > 80%. In terms of sensitivity and specificity, the built model attained the highest sensitivity of 62.26% in the 1–3 age group, and best specificity of 23.53% in the 0–1 age group (Supplementary Table 5). It was worth noting that in children of 0–3 years old, the model only integrating LMR and NLR performed worse than the other two models, only attaining AUC values of 0.68 and 0.64 at the age of 0–1 and 1–3 years old respectively. With the growing age, the model only integrating LMR and NLR significantly outperformed the model with clinical symptoms alone, by attaining AUCs of 0.81 in children over 7 years old. More details could be found in Fig. 5.

Given this multivariate screening model, we calculated the risk scores for each individual in either training or validation sets. Then, by using the cut off values (i.e., the 25 and 75% quantiles) generated from training set, we classified subjects in the validation set into high-risk categories if their risk scores ≥0.763, and identified individuals as medium- or low-risk ones if their risk scores ranged between 0.763 and 0.213, or ≤ 0.213, respectively. Finally, with a total of 1043 individuals in the validation cohort, the multivariate screening model classified 24.07% (251/1043) of them into the high-risk category, with a PPV as high as 87.25%, whereas it identified 52.16% (544/1043) and 23.78% (248/1043) of individuals into the medium and low risk groups of the overall pneumonia, with PPVs of 49.26 and 9.2%, respectively (Table 2). In addition, following the same rationale of using the 25 and 75% quantiles generated from training set as cut-offs, we also classified individuals of the validation set into three risk categories under both the model considering clinical symptoms alone and the model only integrating LMR and NLR respectively. It was worth noting that these two models only reached PPVs of 74.82 and 77.13% in the high-risk category respectively, lower than that of the multivariate screening model. Meanwhile, the model considering signs alone attained the sensitivity as high as 61.76% in the high-risk group, followed by our multivariate screening model and the model only considering LMR and NLR, with sensitivities of 42.94 and 33.73% respectively (Supplementary Table 6 and Supplementary Table 7).

**Table 2:** The performance of multivariate screening model for overall pneumonia in the cohort

	High risk	Medium risk	Low risk	Total
Total	251	544	248	1043
Case	219	268	23	510
PPV	87.25%	49.26%	9.27%	48.90%
Sensitivity	42.94%	52.55%	4.51%	
Specificity	6.00%	51.78%	42.21%	

## Discussion

In this study, we constructed a univariate screening model considering blood biomarkers only and a multivariate screening model that integrated both blood biomarkers and clinical symptoms to identify pneumonia from URTI in children. As validated, the single-variate screening model of LMR and NLR attained AUCs of 0.76 and 0.71 respectively, outperformed other biomarkers (AUC: 0.49–0.69) in regular blood testing on screening accuracy. After further integrating LMR and NLR with three common clinical symptoms of fever, cough and rhinorrhea, the multivariate screening model obtained an increased discriminative ability, reached an AUC of 0.84 for distinguishing pneumonia from URTI, better than using clinical symptoms only (AUC = 0.81). According to the risk scores of the multivariate model, we stratified individuals into three distinct pneumonia-risk categories (high, medium and low), and found that, 87.25% of the pneumonia children were successfully identified by the model as high risk. On the contrary, only 9.27% of the cases were subject to low-risk group.

As an easy and inexpensive routine examination technique, complete blood counts can provides information about WBC, neutrophil, C-reactive protein, monocyte and lymphocyte, therefore, the ratio of neutrophils to lymphocytes (NLR) can be easily calculated [23–25]. Our current study adds to the value of the NLR by showing that this marker is of interest in distinguishing pneumonia from URTI in children, we found that the discriminatory capacity of NLR outweighed predictive values of traditional biomarkers such as CRP, which has been identified elsewhere as a good indicator of CAP risk (community-acquired pneumonia risk) [22]. It has also been reported that, compared with healthy people, NLR was significantly increased in pneumonia, indicating that it can be used as predictors for the presence of pneumonia [26, 27]. A previous study suggested NLR as an indicator to identify adults with pneumonia, which attained an AUC of 0.938 [28], higher than that of our study. The difference in performance may partly due to the heterogeneity of the study population, for they aimed to capture pneumonia cases from healthy adults while we distinguished pneumonia from URTI in children. However, the underlying cause of lower NLR level in pneumonia group than that of URTI has not been yet clear. Lower NLR was defined as increased lymphocyte counts or decreased neutrophils count. Lymphocytes are present in the lung and have been shown to play a role in several lung diseases (pneumonia, asthma, chronic obstructive pulmonary disease and so on) in both humans and mice [29, 30]. More specifically, lymphocytes are recruited to the lung in response to pulmonary infections (Aspergillus fumigatus, Klebsiella pneumonia) [31]. It has been reported that increased lymphocytosis is associated with pulmonary hypertension, pneumonia, and death [32]. Fabienne Venet et al. observed the different lymphocyte subpopulations present and/or recruited to the lung in the development of acute lung injury (ALI), where ALI was mainly caused by pneumonia. Mice data suggested that, during the ALI, the recruiting of CD4+ T lymphocytes to the lung is partly activated by the increased level of IL-16 produced in ECs (lung endothelial cells), whereas the recruiting of neutrophils is inhibited by the increased IL-10 level during these process [33], which supported our observed lower level of NLR in pneumonia children.

Several recent studies suggest that the LMR is an economical, readily available and reproducible test for predicting clinical outcomes of patients with solid tumors and hematological malignancy, including nasopharyngeal carcinoma, colorectal cancer and lymphoma and so on [34–36]. Moreover, Merekoulias et al. found that in 90% of patients who had influenza virus, lymphopenia and/or monocytosis, and LMR could be used as a time-saving and cost-effective screening test for influenza virus infection [37]. Our study proved the potential utility of this infection marker in children to discriminate pneumonia from URTI. It’s worth noting that the discriminatory capacity of the LMR outweighed predictive values of NLR in identifying the overall pneumonia as well as bacterial pneumonia, but lower than that of NLR in the identification of viral pneumonia. The difference may partly due to the relatively small number (460) of children affected by viral pneumonia enrolled in our study, compared with 2088 cases with bacterial pneumonia. Elevated LMR was defined as decreased monocyte or increased lymphocyte counts. However, compared to URTI, the relationship between higher levels of LMR in pneumonia is not yet clear. In acute lung inflammation, blood monocytes migrate into the lung parenchyma and bronchoalveolar space, where they differentiate into “monocyte-derived” alveolar macrophages (Mo-AMs) and orchestrate a proinflammatory and profibrotic response, leading to the decreased level of monocyte count in the blood [38–41]. In the lung, the newly arrived monocytes can enhance microbicidal activities by producing TNF and nitric oxide synthases [42, 43]. Moreover, Yong found that monocyte chemoattractant protein–1 increases in the serum of immunocompetent patients with CAP [44], which may lead to the reduced concentration of monocyte in the blood, as well as the decreased level of LMR comparing to URTI.

In terms of application, we hope this easy-to-implement pneumonia primary screening tool could be utilized in community health centers or resource-poor primary care settings in several ways. First, it may be helpful in triage procedures. For instance, when children come to primary health care centers, their vital signs and blood routine value would be entered into the model and generate a predicted pneumonia risk. These predicted results could help to prioritize which children should be sent for chest radiography examination, and thus would help to ensure that medical resources and equipment is dedicated to children with the highest needs. By triaging patients more effectively, corresponding radiation hazards can be eliminated, and avoidable medical expenses and family burdens caused by excessive medical treatment will ultimately be reduced. Another potential application of this screening tool is to assist primary physicians to improve the accuracy of diagnosis. While formulating a differential diagnosis, community physicians often draw on their past experience and may not have extensive expertise for patients presenting with similar signs, so misdiagnosis may be a distinct possibility in these cases. Using this screening tool, community physicians can use the risk derived from the model to help improve the accuracy of his differential diagnosis, thereby reducing the bias of individual physicians.

Our study has several limitations. First, the bacterial pneumonia and viral pneumonia was not balanced in our data, which might cause bias in terms of risk assessment and performance evaluation. Second, in this study, although pediatricians made a comprehensive diagnosis of URTI based on clinical signs, blood routine tests, and possible chest X-ray results, case of bronchitis, bronchiolitis or wheezing, which were true LRTI, may still be potentially misclassified as URTI, however, such misclassification rate should be low. Third, this study only included three common signs (fever, cough and rhinorrhea) into the screening model, while some important clinical symptoms such as tachypnea were not recorded as structured data in the hospital information system and thus could not be accurately and directly extracted from the big data platform. Since such symptoms are potentially valuable indicators of pneumonia, the missing information may reduce the accuracy of the developed screening tool. Finally, in this study, the bacterial or viral infections were not labeled for upper respiratory tract infection patients in the hospital information system, thus it is hoped that the predictive value and the underlying mechanism of the two indicators, NLR and LMR, in identifying different sub-types of pneumonia and URTI could be further elucidated.

## Conclusion

In conclusion, we have constructed and validated screening models to assess pneumonia risk in children. The multivariate screening model achieved a 0.84 predictive accuracy in validation cohorts, and successfully stratified patients into three distinct pneumonia risk categories. We hope that the constructed pneumonia screening tool for children could be applied and evaluated further in primary health center to explore its utility and cost effectiveness, and ultimately facilitate clinical decision making for children’s pneumonia diagnosis at the community level.

## Supplementary Information

**Additional file 1 Supplementary Figure 1.** The ROC curve for LMR and NLR to differentiate two different types of pneumonia against URTI. The figure shows the subgroup analysis of LMR and NLR in distinguishing URTI from viral or bacterial pneumonia.

**Additional file 2 Supplementary Figure 2.** The ROC curve of three models to identify two different types of pneumonia against URTI, as well as to differentiate bacterial pneumonia from viral pneumonia. The figure shows the subgroup analysis of three models to identify two different types of pneumonia against URTI, as well as to differentiate bacterial pneumonia from viral pneumonia.

**Additional file 3 Supplementary Figure 3.** The ROC curve of combing age, fever, cough, RHI and CRP to identify two different types of pneumonia against URTI. The figure shows the model considering age, fever, cough, RHI and CRP to identify URTI against overall pneumonia, as well as two different types of pneumonia.

**Additional file 4 Supplementary Table 1.** STARD checklist. STARD checklist contains a list of essential items to make sure the report of a diagnostic accuracy study contains the necessary information.

**Additional file 5 Supplementary Table 2.** Diagnostic criteria for URTI and pneumonia. This file contains a table of diagnostic criteria and procedures for URTI and pneumonia, as well as a paragraph to fully explain it.

**Additional file 6 Supplementary Table 3.** AUC values for each blood biomarkers to differentiate pneumonia, viral pneumonia and bacterial pneumonia from URTI. This table shows the AUC values for each blood biomarkers in identifying pneumonia, viral pneumonia and bacterial pneumonia against URTI.

**Additional file 7 Supplementary Table 4.** Correlation between LMR, NLR and three different types of pneumonia. This table shows the relationship between LMR, NLR and pneumonia.

**Additional file 8 Supplementary Table 5.** The distribution of high-risk children across the different age groups, identified by the built multivariate screening model. This table shows PPVs, sensitivity and specificity of high-risk children identified by the built multivariate screening model across various age stratifications.

**Additional file 9 Supplementary Table 6.** The performance of the model considering clinical signs alone for overall pneumonia in the cohort. This table shows PPVs, sensitivity and specificity of the model considering clinical signs alone for overall pneumonia.

**Additional file 10 Supplementary Table 7.** The performance of the model only considering LMR and NLR for overall pneumonia in the cohort. This table shows PPVs, sensitivity and specificity of the model only considering LMR and NLR for overall pneumonia.

## Data Availability

The datasets used during the current study are available from the corresponding author on reasonable request.

## Abbreviations

URTI: Upper Respiratory Tract Infection
LMR: Lymphocyte-To-Monocyte Ratio
NLR: Neutrophil-To-Lymphocyte Ratio
STARD: Standard for Reporting of Diagnostic Accuracy Studies
NC: Neutrophils Cell
MC: Monocytes Cell
LC: Lymphocyte Cell
WBC: White Blood Cell Count
CRP: C-Reactive protein
PCR: Polymerase chain reaction
RHI: Rhinorrhea
GGO: Ground-glass opacity
RSV: Respiratory Syncytial Virus
ADVDNA: Adenovirus Nucleic Acid
FluA-Ag: Influenza A virus Antigen
PPVs: Positive predictive values
