# Supplementary material for: The value of lymphocyte-to-monocyte ratio and neutrophil-to-lymphocyte ratio in differentiating pneumonia from upper respiratory tract infection (URTI) in children: a cross-sectional study
Source: BMC Pediatr. 2021 Dec 3;21:545. doi: 10.1186/s12887-021-03018-y (PMC8641150; doi:10.1186/s12887-021-03018-y)
Supplement: Supplementary file 1 — Additional file 1 Supplementary Figure 1. The ROC curve for LMR and NLR to differentiate two different types of pneumonia against URTI. The figure shows the subgroup analysis of LMR and NLR in distinguishing URTI from viral or bacterial pneumonia. [file 12887_2021_3018_MOESM1_ESM.docx]

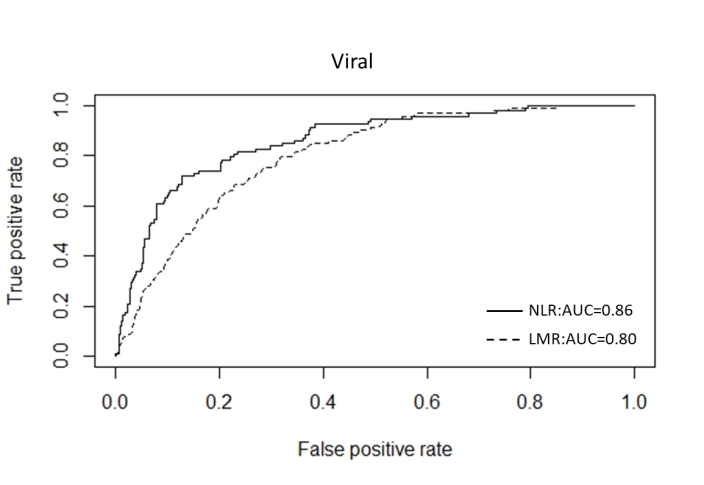

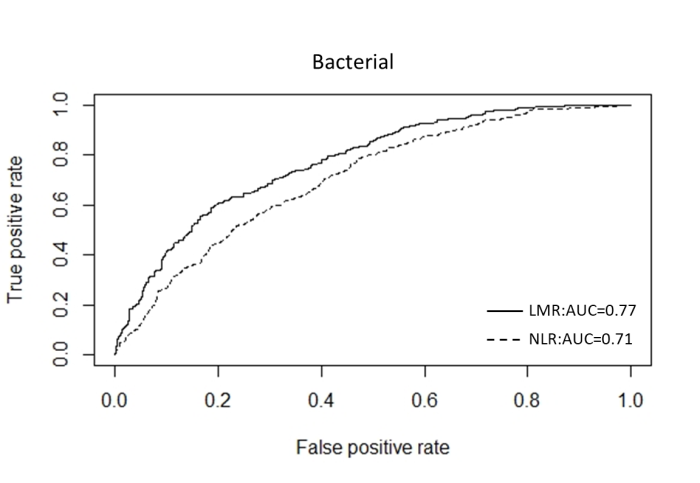


**Supplementary Figure 1. The ROC curve for LMR and NLR to differentiate two different types of pneumonia against URTI**
