# Supplementary material for: The value of lymphocyte-to-monocyte ratio and neutrophil-to-lymphocyte ratio in differentiating pneumonia from upper respiratory tract infection (URTI) in children: a cross-sectional study
Source: BMC Pediatr. 2021 Dec 3;21:545. doi: 10.1186/s12887-021-03018-y (PMC8641150; doi:10.1186/s12887-021-03018-y)
Supplement: Supplementary file 2 — Additional file 2 Supplementary Figure 2. The ROC curve of three models to identify two different types of pneumonia against URTI, as well as to differentiate bacterial pneumonia from viral pneumonia. The figure shows the subgroup analysis of three models to identify two different types of pneumonia against URTI, as well as to differentiate bacterial pneumonia from viral pneumonia. [file 12887_2021_3018_MOESM2_ESM.docx]

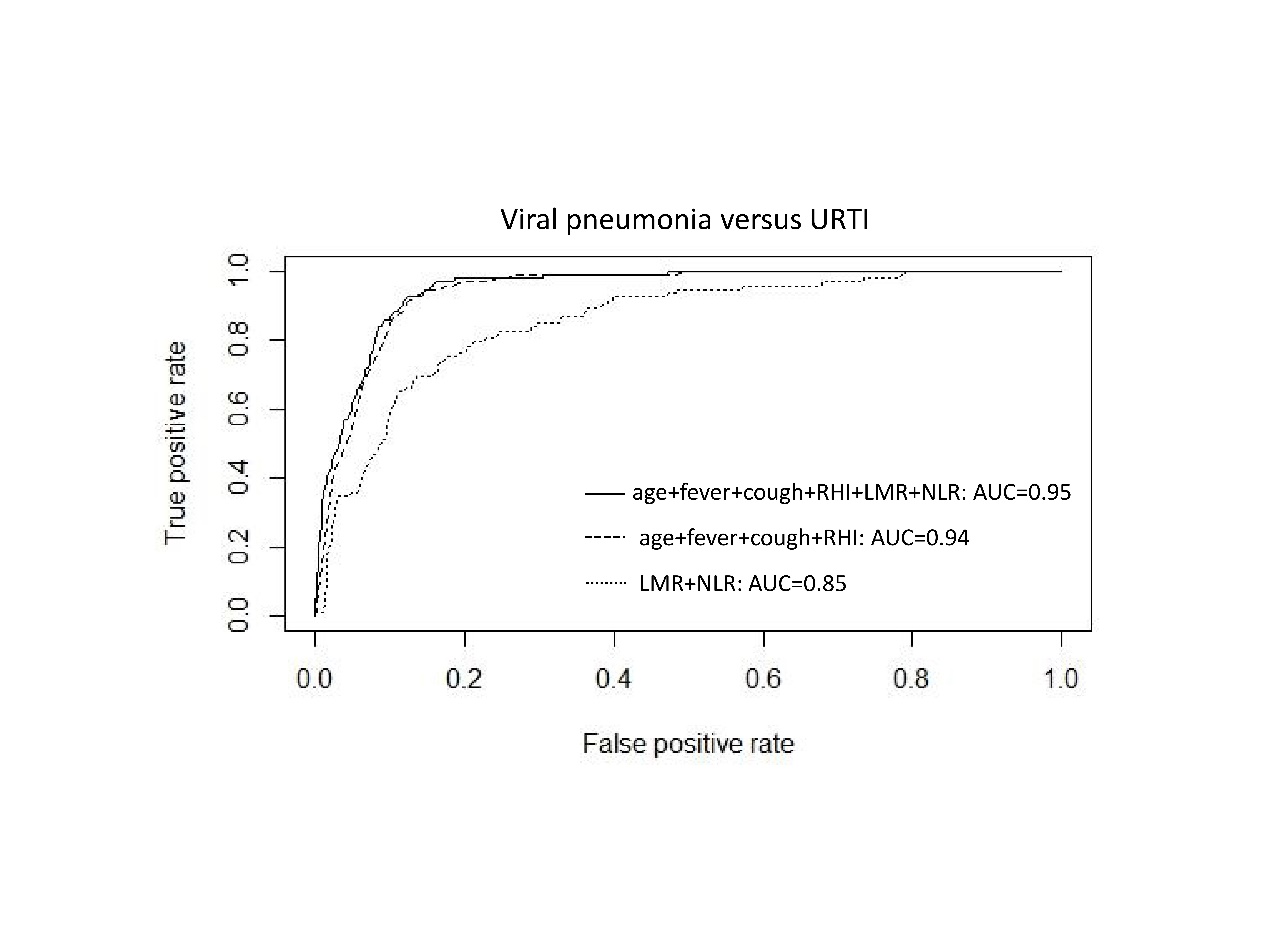

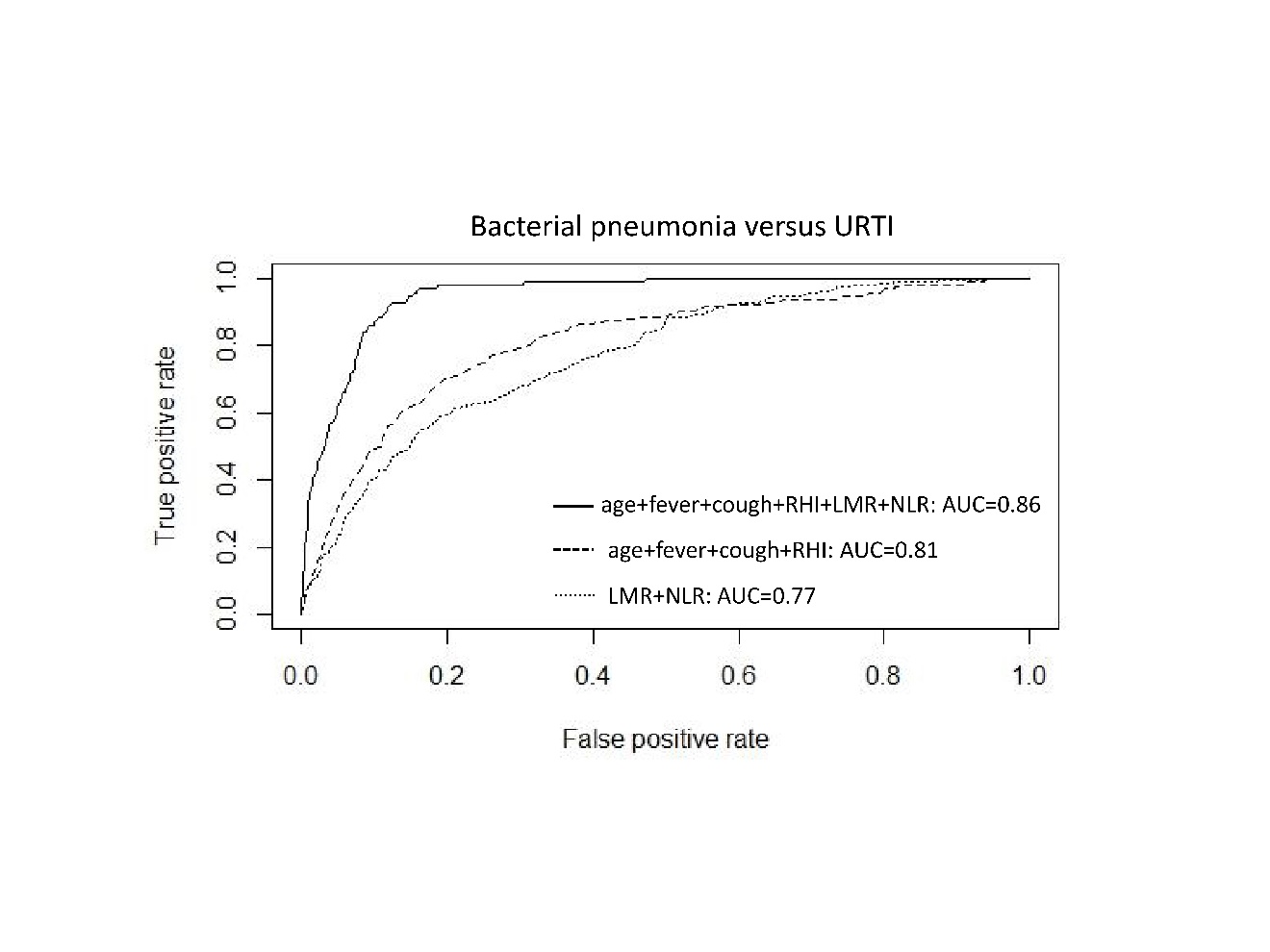

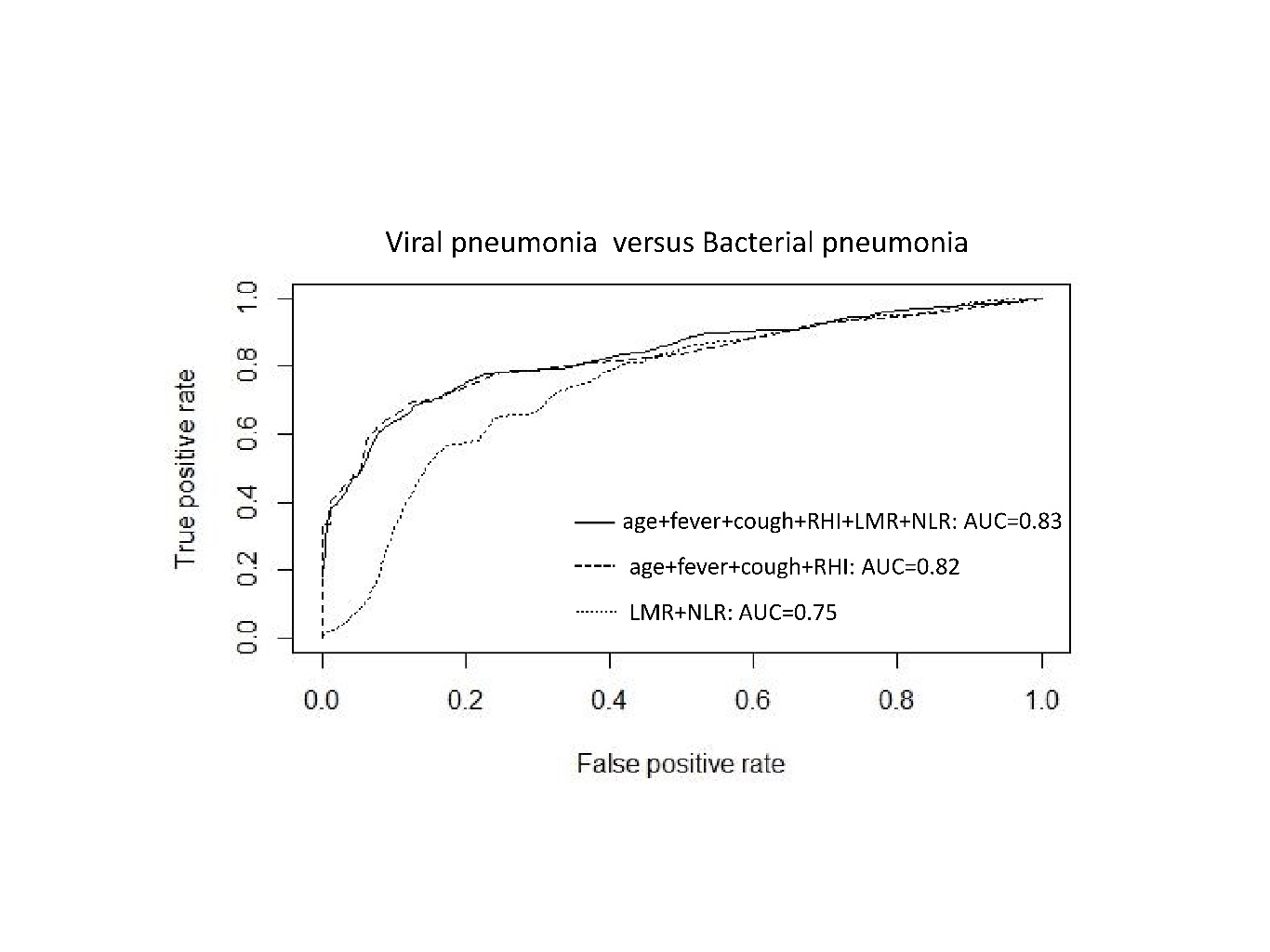


**Supplementary Figure 2. The ROC curve of three models to identify two different types of pneumonia against URTI, as well as to differentiate bacterial pneumonia from viral pneumonia.**
