# Supplementary material for: The value of lymphocyte-to-monocyte ratio and neutrophil-to-lymphocyte ratio in differentiating pneumonia from upper respiratory tract infection (URTI) in children: a cross-sectional study
Source: BMC Pediatr. 2021 Dec 3;21:545. doi: 10.1186/s12887-021-03018-y (PMC8641150; doi:10.1186/s12887-021-03018-y)
Supplement: Supplementary file 3 — Additional file 3 Supplementary Figure 3. The ROC curve of combing age, fever, cough, RHI and CRP to identify two different types of pneumonia against URTI. The figure shows the model considering age, fever, cough, RHI and CRP to identify URTI against overall pneumonia, as well as two different types of pneumonia. [file 12887_2021_3018_MOESM3_ESM.docx]

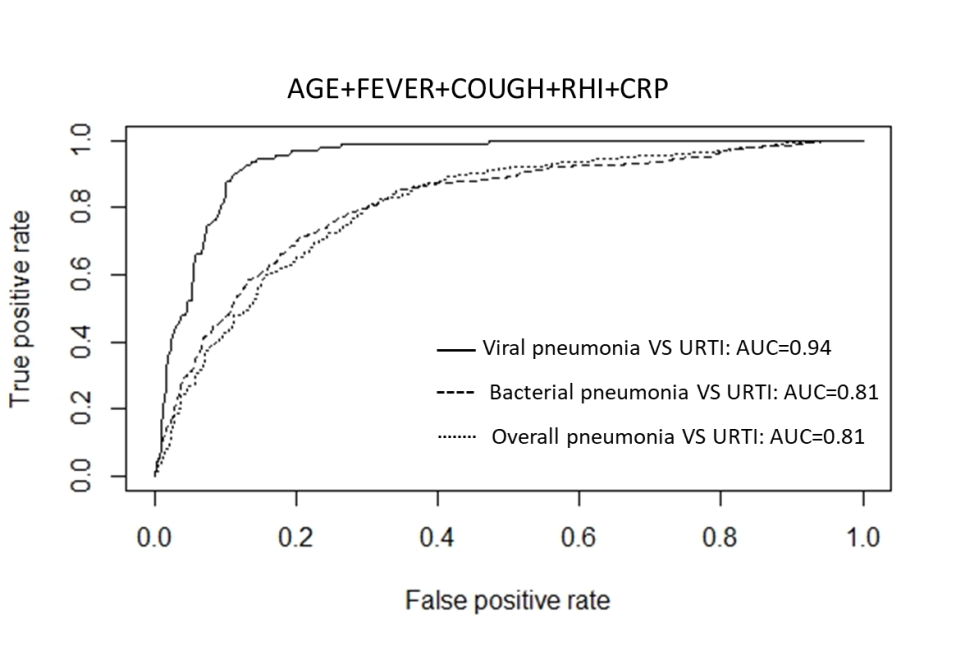


**Supplementary Figure 3. The ROC curve of combing age, fever, cough, RHI and CRP to identify two different types of pneumonia against URTI**
