# Supplementary material for: The value of lymphocyte-to-monocyte ratio and neutrophil-to-lymphocyte ratio in differentiating pneumonia from upper respiratory tract infection (URTI) in children: a cross-sectional study
Source: BMC Pediatr. 2021 Dec 3;21:545. doi: 10.1186/s12887-021-03018-y (PMC8641150; doi:10.1186/s12887-021-03018-y)
Supplement: Supplementary file 4 — Additional file 4 Supplementary Table 1. STARD checklist. STARD checklist contains a list of essential items to make sure the report of a diagnostic accuracy study contains the necessary information. [file 12887_2021_3018_MOESM4_ESM.docx]

**Supplementary Table 1. STARD checklist**

| **Section & Topic** | **No** | **Item** | **Reported on page #** |
| --- | --- | --- | --- |
|  |  |  |  |
| **TITLE OR ABSTRACT** |  |  |  |
|  | **1** | Identification as a study of diagnostic accuracy using at least one measure of accuracy (such as sensitivity, specificity, predictive values, or AUC) | 3 |
| **ABSTRACT** |  |  |  |
|  | **2** | Structured summary of study design, methods, results, and conclusions (for specific guidance, see STARD for Abstracts) | 3-4 |
| **INTRODUCTION** |  |  |  |
|  | **3** | Scientific and clinical background, including the intended use and clinical role of the index test | 5-6 |
|  | **4** | Study objectives and hypotheses | 6 |
| **METHODS** |  |  |  |
| *Study design* | **5** | Whether data collection was planned before the index test and reference standard were performed (prospective study) or after (retrospective study) | 7 |
| *Participants* | **6** | Eligibility criteria | 7-9 |
|  | **7** | On what basis potentially eligible participants were identified (such as symptoms, results from previous tests, inclusion in registry) | 7-9 |
|  | **8** | Where and when potentially eligible participants were identified (setting, location and dates) | 7 |
|  | **9** | Whether participants formed a consecutive, random or convenience series | 7 |
| *Test methods* | **10a** | Index test, in sufficient detail to allow replication | 10-11 |
|  | **10b** | Reference standard, in sufficient detail to allow replication | 7-9, and Supplementary Table 2 |
|  | **11** | Rationale for choosing the reference standard (if alternatives exist) | Supplementary Table 2 |
|  | **12a** | Definition of and rationale for test positivity cut-offs or result categories of the index test, distinguishing pre-specified from exploratory | 11 and 17 |
|  | **12b** | Definition of and rationale for test positivity cut-offs or result categories of the reference standard, distinguishing pre-specified from exploratory | Supplementary Table 2 |
|  | **13a** | Whether clinical information and reference standard results were available to the performers/readers of the index test | Supplementary Table 2 |
|  | **13b** | Whether clinical information and index test results were available to the assessors of the reference standard | Supplementary Table 2 |
| *Analysis* | **14** | Methods for estimating or comparing measures of diagnostic accuracy | 10-11 |
|  | **15** | How indeterminate index test or reference standard results were handled | 9 |
|  | **16** | How missing data on the index test and reference standard were handled | 9 |
|  | **17** | Any analyses of variability in diagnostic accuracy, distinguishing pre-specified from exploratory | 10-11 |
|  | **18** | Intended sample size and how it was determined | 7 |
| **RESULTS** |  |  |  |
| *Participants* | **19** | Flow of participants, using a diagram | 9 |
|  | **20** | Baseline demographic and clinical characteristics of participants | 11-12 |
|  | **21a** | Distribution of severity of disease in those with the target condition | Supplementary Table 2 |
|  | **21b** | Distribution of alternative diagnoses in those without the target condition | Supplementary Table 2 |
|  | **22** | Time interval and any clinical interventions between index test and reference standard | 7 |
| *Test results* | **23** | Cross tabulation of the index test results (or their distribution) by the results of the reference standard | 18, Supplementary Table 6, and Supplementary Table 7 |
|  | **24** | Estimates of diagnostic accuracy and their precision (such as 95% confidence intervals) | 13-18 |
|  | **25** | Any adverse events from performing the index test or the reference standard | None |
| **DISCUSSION** |  |  |  |
|  | **26** | Study limitations, including sources of potential bias, statistical uncertainty, and generalizability | 22 |
|  | **27** | Implications for practice, including the intended use and clinical role of the index test | 21-22 |
| **OTHER INFORMATION** |  |  |  |
|  | **28** | Registration number and name of registry | 7 |
|  | **29** | Where the full study protocol can be accessed | 23 |
|  | **30** | Sources of funding and other support; role of funders | 24 |
|  |  |  |  |
