# Supplementary material for: The value of lymphocyte-to-monocyte ratio and neutrophil-to-lymphocyte ratio in differentiating pneumonia from upper respiratory tract infection (URTI) in children: a cross-sectional study
Source: BMC Pediatr. 2021 Dec 3;21:545. doi: 10.1186/s12887-021-03018-y (PMC8641150; doi:10.1186/s12887-021-03018-y)
Supplement: Supplementary file 5 — Additional file 5 Supplementary Table 2. Diagnostic criteria for URTI and pneumonia. This file contains a table of diagnostic criteria and procedures for URTI and pneumonia, as well as a paragraph to fully explain it. [file 12887_2021_3018_MOESM5_ESM.docx]

Making use of the revised WHO classification for pneumonia (WHO, 2014) and Chinese guidelines (2013) for the management of pneumonia in children[1, 2], pediatricians made diagnosis of each patient according to the following procedure: Initially, through interrogation and physical examinations, all inpatients and outpatients’ clinical symptoms and medical history were collected. Specifically, patients with signs of cough, body temperature>37℃, no abnormalities in auscultation etc. were preliminarily diagnosed with URTI, while patients who had symptoms of cough, body temperature between 37℃ and 38.5℃, wheezing etc. were suspected of viral pneumonia, and those with symptoms such as cough, temperature≥38.5℃, rhonchus or moist rales etc., were initially diagnosed with bacterial pneumonia. Then, all patients were sent for blood routine tests. Patients who suffered from bacterial infections, either as URTI or LRTI, generally showed increased WBC and neutrophils ratio, while patients with suspected viral infections usually have normal or low WBC and increased lymphocyte ratio. At the same time, patients with suspected pneumonia were sent for chest X-ray examinations to see if there were any abnormalities or lesions in their lungs. Combining all the above results, patients with symptoms or signs of URTI but no abnormalities in pulmonary images (if any) were finally diagnosed with URTI. Meanwhile, patients who had multifocal 1–10-mm well-defined or ill-defined nodular opacity with a surrounding halo or patchy ground-glass opacity (GGO), or other described features, were suspected of having viral pneumonia, while patients who had features of patchy shadow, lung consolidation or centrilobular lung nodules, etc., were considered for bacterial pneumonia. Following that, patients with suspected viral pneumonia went for a nasopharyngeal or throat swab to detect if any of 8 viruses were presented. On the other hand, those who were suspected of having bacterial pneumonia went for other tests to detect if any of 13 bacteria could be observed or cultured in nasopharyngeal swab, sputum culture, alveolar lavage fluid, fiberoptic bronchoscopy smear or pleural effusion. Finally, combining with clinical symptoms, medical history, and results from the described laboratory, radiographic and pathogenic tests, the pediatrician made a diagnosis of each patient. More details about URTI and pneumonia diagnosis could be found in **Supplementary Table 2.**

Supplementary Table 2. Diagnostic criteria for URTI and pneumonia

|  | **URTI** | **Viral pneumonia** | **Bacterial pneumonia** |
| --- | --- | --- | --- |
| **Clinical symptoms and medical history** | - cough - body temperature>37℃ - no abnormalities in auscultation - swollen and congested tonsils - hyperemia, edema, and secretions in nasal mucosa or pharynx - runny nose - nasal congestion - headache - sneezing | - cough - body temperature between 37℃ and 38.5℃ - wheezing - tachypnoea - breathlessness - chest pain | - cough - body temperature≥38.5℃ - rhonchus or moist rales - tachypnea - breathlessness - chest pain |
| **Blood routine test** | normal or low WBC and increased lymphocyte ratio in viral infection, increased WBC and neutrophils ratio in bacterial infection | normal or low WBC and increased lymphocyte ratio | increased WBC and neutrophils ratio |
| **Chest X-ray** | no pulmonary imaging changes | multifocal 1–10-mm well-defined or ill-defined nodular opacity with a surrounding halo or patchy ground-glass opacity (GGO), diffuse ill-defined patchy GGO with interlobular septal thickening, multiple ill-defined nodules or GGO along the bronchovascular bundles, multiple irregular areas of consolidation along the bronchovascular bundles and diffuse GGO with interlobular septal thickening, or multiple ill-defined patchy areas of GGO with interlobular septal thickening in both lungs. | patchy shadow, lung consolidation(usually lobed or segmentary with bronchial inflation), centrilobular lung nodules (Solid or mixed density nodules along the bronchovascular bundle), or tree-in-bud sign(linear branching shadow), alveolar infiltrates, "round" pneumonia, or with complications of pleural effusion/empyema, lung abscess, necrotizing pneumonia, pneumatocele |
| **Pathogenic detection** |  | **Nasopharyngeal swab:** respiratory syncytial virus (RSV), Adenovirus nucleic acid (ADVDNA), influenza A virus antigen (FluA-Ag), parainfluenza virus type 1, parainfluenza virus type 2, parainfluenza virus type 3; | **Nasopharyngeal swab:** staphylococcus hominis, proteus mirabilis, staphylococcus aureus, klebsiella pneumoniae, or candida albicans |
|  |  | **Throat swabs:** H7N9 virus, influenza A virus antigen (FluA-Ag), influenza B virus antigen (FluB-Ag) | **Sputum culture:** pseudomonas aeruginosa, candida albicans, mixed flora, klebsiella pneumoniae, staphylococcus aureus, acinetobacter baumannii, klebsiella pneumoniae subspecies, serratia marcescens, proteus mirabilis, a large number of gram-negative (G-) bacteria, burkholderia cepacia |
|  |  |  | **Alveolar lavage fluid:** pseudomonas aeruginosa |
|  |  |  | **Fiberoptic bronchoscopy smear:** pseudomonas aeruginosa, candida albicans, acinetobacter baumannii, klebsiella pneumoniae subspecies, a large number of gram-negative (G-) bacteria or acid-fast bacilli (++) |
|  |  |  | **Pleural effusion:** pseudomonas aeruginosa, staphylococcus aureus, acinetobacter baumannii, klebsiella pneumoniae, acinetobacter |

*References:*

1. World Health Organization. Revised WHO Classification and Treatment of Childhood Pneumonia at Health Facilities: Evidence Summaries. 2014.

2. Chang-chong li, Yun-xiao shang, Xu-zhuang shen, Chen zm S zhao. Guidelines for the Management of Community-acquired Pneumonia in Children (2013 Revised). Chinese J Pediatr. 2013;51:745–52.
