# Supplementary material for: The value of lymphocyte-to-monocyte ratio and neutrophil-to-lymphocyte ratio in differentiating pneumonia from upper respiratory tract infection (URTI) in children: a cross-sectional study
Source: BMC Pediatr. 2021 Dec 3;21:545. doi: 10.1186/s12887-021-03018-y (PMC8641150; doi:10.1186/s12887-021-03018-y)
Supplement: Supplementary file 6 — Additional file 6 Supplementary Table 3. AUC values for each blood biomarkers to differentiate pneumonia, viral pneumonia and bacterial pneumonia from URTI. This table shows the AUC values for each blood biomarkers in identifying pneumonia, viral pneumonia and bacterial pneumonia against URTI. [file 12887_2021_3018_MOESM6_ESM.docx]

**Supplementary Table 3. AUC values for each blood biomarkers to differentiate pneumonia, viral pneumonia and bacterial pneumonia from URTI**

|  |  | NC | MC | LC | WBC | CRP | LMR | NLR |
| --- | --- | --- | --- | --- | --- | --- | --- | --- |
| Pneumonia | Train | 0.66 | 0.60 | 0.72 | 0.50 | 0.48 | 0.77 | 0.74 |
|  | Test | 0.63 | 0.60 | 0.69 | 0.51 | 0.49 | 0.76 | 0.71 |
| Viral | Train | 0.76 | 0.55 | 0.87 | 0.57 | 0.57 | 0.80 | 0.87 |
|  | Test | 0.76 | 0.56 | 0.85 | 0.54 | 0.54 | 0.80 | 0.86 |
| Bacterial | Train | 0.64 | 0.63 | 0.69 | 0.51 | 0.49 | 0.76 | 0.71 |
|  | Test | 0.63 | 0.66 | 0.69 | 0.52 | 0.50 | 0.77 | 0.71 |
