# Supplementary material for: The value of lymphocyte-to-monocyte ratio and neutrophil-to-lymphocyte ratio in differentiating pneumonia from upper respiratory tract infection (URTI) in children: a cross-sectional study
Source: BMC Pediatr. 2021 Dec 3;21:545. doi: 10.1186/s12887-021-03018-y (PMC8641150; doi:10.1186/s12887-021-03018-y)
Supplement: Supplementary file 7 — Additional file 7 Supplementary Table 4. Correlation between LMR, NLR and three different types of pneumonia. This table shows the relationship between LMR, NLR and pneumonia. [file 12887_2021_3018_MOESM7_ESM.docx]

**Supplementary Table 4. Correlation between LMR, NLR and three different types of pneumonia**

|  | LMR | | NLR | |
| --- | --- | --- | --- | --- |
|  | *β* | OR | *β* | OR |
| Combined | 0.24 | 1.27 | -0.40 | 0.67 |
| Viral | 0.22 | 1.24 | -1.48 | 0.23 |
| Bacterial | 0.23 | 1.26 | -0.32 | 0.73 |
