# Supplementary material for: The value of lymphocyte-to-monocyte ratio and neutrophil-to-lymphocyte ratio in differentiating pneumonia from upper respiratory tract infection (URTI) in children: a cross-sectional study
Source: BMC Pediatr. 2021 Dec 3;21:545. doi: 10.1186/s12887-021-03018-y (PMC8641150; doi:10.1186/s12887-021-03018-y)
Supplement: Supplementary file 8 — Additional file 8 Supplementary Table 5. The distribution of high-risk children across the different age groups, identified by the built multivariate screening model. This table shows PPVs, sensitivity and specificity of high-risk children identified by the built multivariate screening model across various age stratifications. [file 12887_2021_3018_MOESM8_ESM.docx]

**Supplementary Table 5. The distribution of high-risk children across the different age groups, identified by the built multivariate screening model**

| Age | Total | Cases | Total captured in high-risk category | Case captured in high-risk category | PPV | Sensitivity | Specificity |
| --- | --- | --- | --- | --- | --- | --- | --- |
| 0-1 | 188 | 137 | 94 | 82 | 87.23% | 59.85% | 23.53% |
| 1-3 | 189 | 106 | 79 | 66 | 83.54% | 62.26% | 15.66% |
| 3-5 | 187 | 109 | 47 | 42 | 89.36% | 38.53% | 6.41% |
| 5-7 | 153 | 57 | 18 | 17 | 94.44% | 29.82% | 1.04% |
| 7-18 | 326 | 101 | 13 | 12 | 92.31% | 11.88% | 0.44% |
