# Supplementary material for: The value of lymphocyte-to-monocyte ratio and neutrophil-to-lymphocyte ratio in differentiating pneumonia from upper respiratory tract infection (URTI) in children: a cross-sectional study
Source: BMC Pediatr. 2021 Dec 3;21:545. doi: 10.1186/s12887-021-03018-y (PMC8641150; doi:10.1186/s12887-021-03018-y)
Supplement: Supplementary file 9 — Additional file 9 Supplementary Table 6. The performance of the model considering clinical signs alone for overall pneumonia in the cohort. This table shows PPVs, sensitivity and specificity of the model considering clinical signs alone for overall pneumonia. [file 12887_2021_3018_MOESM9_ESM.docx]

**Supplementary Table 6. The performance of the model considering clinical signs alone for overall pneumonia in the cohort**

|  | High risk | Medium risk | Low risk | Total |
| --- | --- | --- | --- | --- |
| Total | 421 | 355 | 267 | 1043 |
| Case | 315 | 166 | 29 | 510 |
| PPV | 74.82% | 46.76% | 10.86% | 48.90% |
| Sensitivity | 61.76% | 32.55% | 5.69% |  |
| Specificity | 19.89% | 35.46% | 44.65% |  |
