# Supplementary material for: The value of lymphocyte-to-monocyte ratio and neutrophil-to-lymphocyte ratio in differentiating pneumonia from upper respiratory tract infection (URTI) in children: a cross-sectional study
Source: BMC Pediatr. 2021 Dec 3;21:545. doi: 10.1186/s12887-021-03018-y (PMC8641150; doi:10.1186/s12887-021-03018-y)
Supplement: Supplementary file 10 — Additional file 10 Supplementary Table 7. The performance of the model only considering LMR and NLR for overall pneumonia in the cohort. This table shows PPVs, sensitivity and specificity of the model only considering LMR and NLR for overall pneumonia. [file 12887_2021_3018_MOESM10_ESM.docx]

**Supplementary Table 7. The performance of the model only considering LMR and NLR for overall pneumonia in the cohort**

|  | High risk | Medium risk | Low risk | Total |
| --- | --- | --- | --- | --- |
| Total | 223 | 577 | 243 | 1043 |
| Case | 172 | 290 | 48 | 510 |
| PPV | 77.13% | 50.26% | 19.75% | 48.90% |
| Sensitivity | 33.73% | 56.86% | 9.41% |  |
| Specificity | 9.57% | 53.85% | 36.59% |  |
